# Supplementary material for: Modulation of the liver immune microenvironment by the adeno-associated virus serotype 8 gene therapy vector
Source: Mol Ther Methods Clin Dev. 2020 Nov 4;20:95–108. doi: 10.1016/j.omtm.2020.10.023 (PMC7750493; doi:10.1016/j.omtm.2020.10.023)
Supplement: Document S1. Figure S1 [file mmc1.pdf]

OMTM, Volume *20*

Supplemental Information

Modulation of the liver immune microenvironment by the adeno-associated virus serotype 8 gene therapy vector

Agostina Carestia, Seok-Joo Kim, Franziska Horling, Hanspeter Rottensteiner, Christian Lubich, Birgit M. Reipert, Brian A. Crowe, and Craig N. Jenne

Supplemental Material

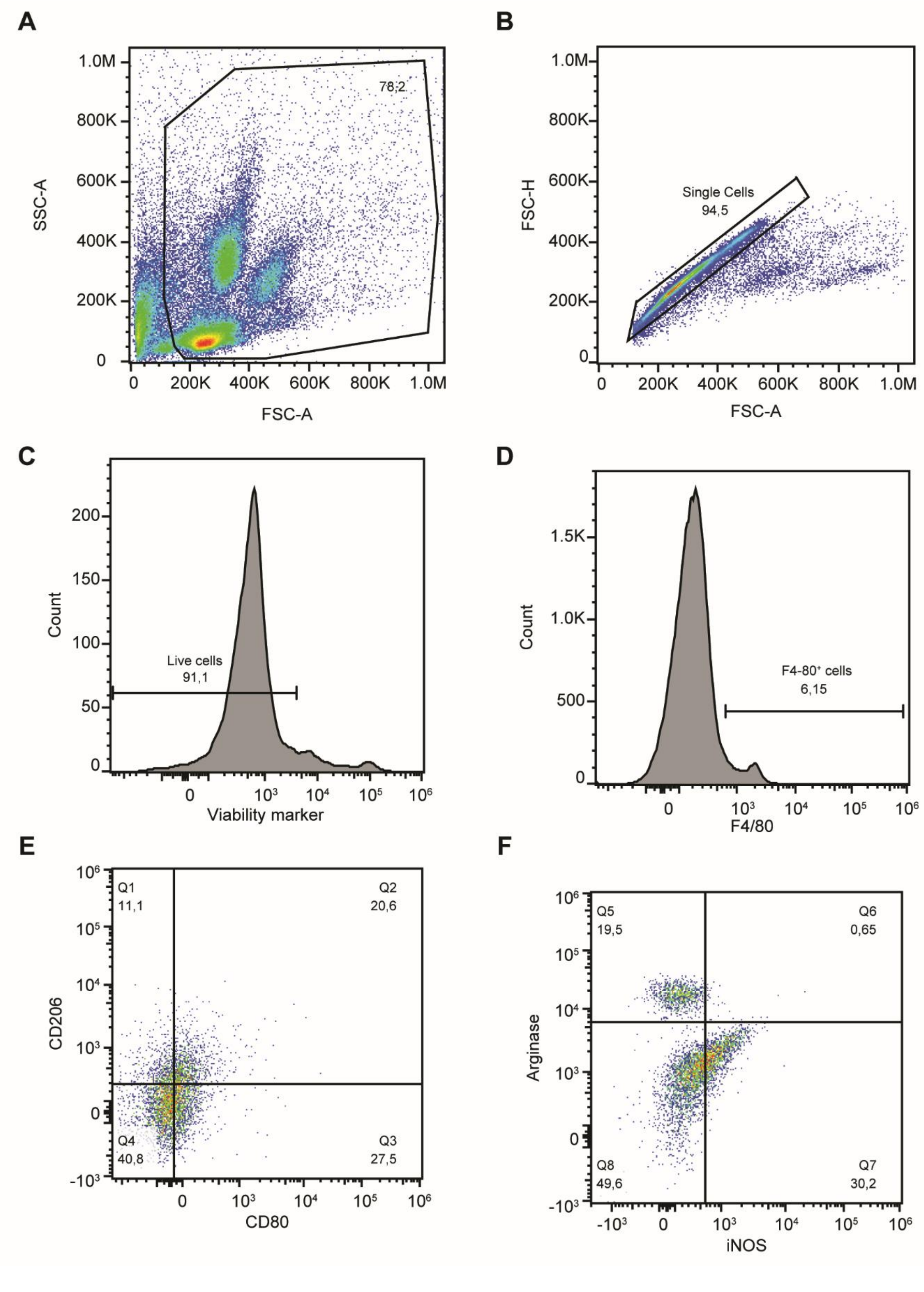

Supplemental Figure 1. Flow cytometry gating strategy used to determine macrophage polarization. After exclusion of debris **(A)** and doublets **(B)**, a live/dead staining was used to exclude dead cells **(C)**. Macrophages were identified on the expression of F4/80 **(D)**. For extracellular staining, a dot plot comparing CD80 and CD206 markers was used **(E)**. For intracellular staining, a dot plot comparing iNOS and Arginase was used

Supplemental Video 1. Viral capture by Kupffer cells and sinusoidal endothelium immediately following i.v. injection of AAV8.

Supplemental Video 2. Accumulation of virus on the surface of Kupffer cells and hepatocytes 30 min post i.v. injection of AAV8.

Supplemental Video 3. *In vivo* propidium iodide labelling of dead/damaged cells in the liver of control mice and AAV8 treated mice 1 day post-infection.

Supplemental Video 4. Imaging of platelet dynamics in the liver AAV8 treated mice 1 day and 3 days post-infection.

Supplemental Video 5. Tracking CD8+ cell interactions with liver Kupffer cells in control mice and at 28 days post AAV8 infection.
