# Supplementary material for: Modulation of the liver immune microenvironment by the adeno-associated virus serotype 8 gene therapy vector
Source: Mol Ther Methods Clin Dev. 2020 Nov 4;20:95–108. doi: 10.1016/j.omtm.2020.10.023 (PMC7750493; doi:10.1016/j.omtm.2020.10.023)
Supplement: Document S2. Article plus supplemental information [file mmc7.pdf]

Molecular Therapy
Methods & Clinical Development
Original Article

Modulation of the liver immune microenvironment by the adeno-associated virus serotype 8 gene therapy vector

Agostina Carestia,[1,4] Seok-Joo Kim,[1,4] Franziska Horling,[2] Hanspeter Rottensteiner,[2] Christian Lubich,[3] Birgit M. Reipert,[2] Brian A. Crowe,[2] and Craig N. Jenne[1]

[1]Department of Microbiology, Immunology, and Infectious Diseases, University of Calgary, Calgary, AB T2N 4N1, Canada; [2]Drug Discovery Austria, Baxalta Innovations GmbH, Vienna, Austria; [3]Institute Krems Bioanalytics, IMC FH Krems, University of Applied Sciences, Krems, Austria

Adeno-associated viruses (AAVs) are emerging as one of the vehicles of choice for gene therapy. However, the potential immunogenicity of these vectors is a major limitation of their use, leading to the necessity of a better understanding of how viral vectors engage the innate immune system. In this study, we demonstrate the immune response mediated by an AAV vector in a mouse model. Mice were infected intravenously with 4 $\times$ 10^{12} copies (cp)/kg of AAV8, and the ensuing immune response was analyzed using intravital microscopy during a period of weeks. Administration of AAV8 resulted in the infection of hepatocytes, and this infection led to a moderate, but significant, activation of the immune system in the liver. This host immune response involved platelet aggregation, neutrophil extracellular trap (NET) formation, and the recruitment of monocytes, B cells, and T cells. The resident liver macrophage population, Kupffer cells, was necessary to initiate this immune response, as its depletion abrogated platelet aggregation and NET formation and delayed the recruitment of immune cells. Moreover, the death of liver cells produced by this AAV was moderate and failed to result in a robust, sustained inflammatory response. Altogether, these data suggest that AAV8 is a suitable vector for gene therapy approaches.

INTRODUCTION

Inherited diseases account for more than 70% of admissions to children's hospitals and 10% of admissions to adult hospitals.[1] Often these genetic diseases require life-long therapy and represent a significant cost both to the health care system and to society as a whole. One of the most promising and best studied strategies for long-term treatment of this family of disorders is gene therapy, a technique that involves the delivery of functional genes into cells in an effort to complement or replace missing or defective genes to correct/ameliorate the disorder.[2,3] Adeno-associated viruses (AAVs) are emerging as one of the vehicles of choice for therapeutic gene transfer.[4,5] AAVs belong to the Parvoviridae family and are composed of a single-stranded DNA genome of approximately 4.7 kb encapsulated in an icosahedral viral capsid.[6]

AAVs thrive as a potential delivery vehicle due to five specific features that promote safe and efficient gene delivery: (1) certain strains of AAVs have not been associated with serious disease;[7,8] (2) AAVs can be engineered to essentially lack viral DNA; (3) they can stably express many genes *in vivo* while inducing a limited immune response to the vector or transgene;[9] (4) they have a wide and promiscuous tropism;[10] and (5) they can achieve efficient and long-lived gene transfer.[4] Taken together, these features elucidate the capacity of AAVs as a gene therapy vehicle.

Although AAVs are among the most promising approaches for the treatment of inherited and acquired diseases, and considerable progress has been made on their use, some complications have arisen. First, an AAV is only capable of efficiently packaging about 5 kb of DNA,[11] excluding many therapeutic genes and approaches from development. Second, because of their non-integrative nature, treatments of pediatric patients with this gene therapy vector require re-administration of the AAV as the organs grow, resulting in a significant dilution of the vector over time.[12] Third, and perhaps the most important, is the fact that many AAV serotypes are endemic within some populations, resulting in extensive pre-existing anti-viral immunity in some patients.[13–15] Although some of these issues can be solved through re-engineering the viral capsid, the acute, innate immune response to viral infection remains a potential barrier. To better understand how viral vectors engage the innate immune system, and how these infections may lead to reprogramming of the tissue microenvironment, we mapped the immune response to AAV8 infection *in vivo* using the approach of intravital microscopy (IVM).

In this study, we demonstrate the immune response mediated by an AAV8 construct expressing enhanced yellow fluorescent protein

Received 6 July 2020; accepted 28 October 2020;
https://doi.org/10.1016/j.omtm.2020.10.023.

[4]These authors contributed equally

Correspondence: Craig N. Jenne, Department of Microbiology, Immunology, and Infectious Diseases, University of Calgary, 3330 Hospital Drive NW, Calgary, AB T2N 4N1, Canada.
E-mail: cnjenne@ucalgary.ca

This is an open access article under the CC BY license (http://creativecommons.org/licenses/by/4.0/).

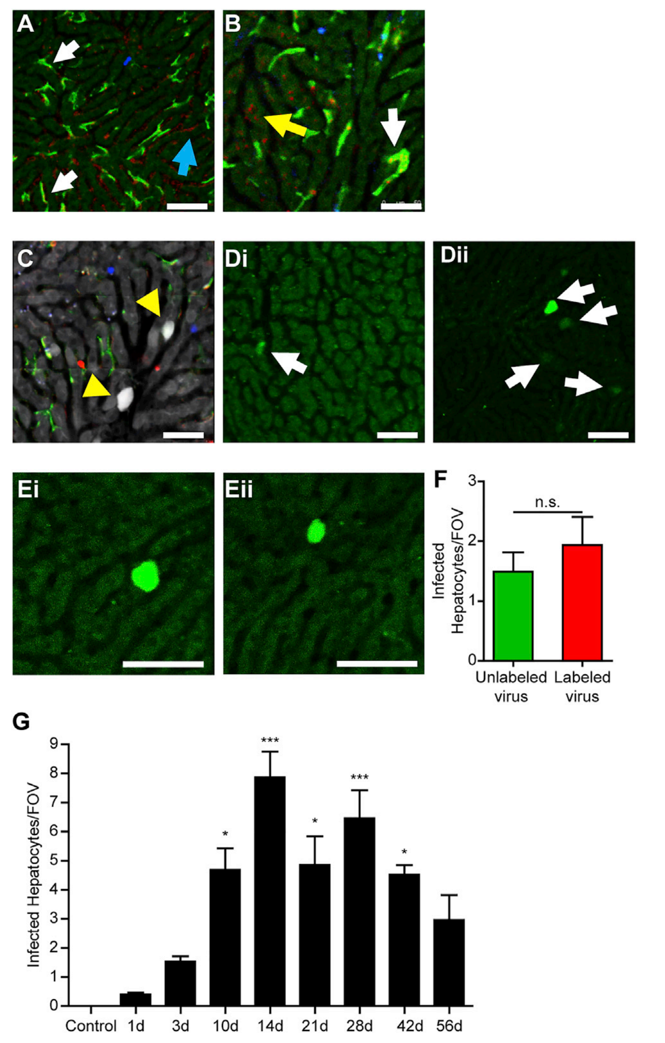

Figure 1. Infection of the liver by AAV8

(A and B) Visualization of virus capture by liver cells following injection of labeled AAV8 10 min post-injection (A) and 30 min post-injection (B). Hepatocytes are autofluorescent in dark green, Kupffer cells (F4/80$^+$) are bright green, neutrophils (Ly6G$^+$) are blue, and Alexa Fluor-labeled virus is red. Virus associated with endothelium is denoted with a blue arrow, virus on Kupffer cells is denoted with white arrows, and virus on hepatocytes is denoted with a yellow arrow. (C) Infected hepatocytes labeled by expression of a virus-delivered eYFP gene (denoted with yellow arrowheads) were visualized by IVM 48 h post-infection. Kupffer cells are labeled in green, CD8$^+$ cells are labeled in blue, and CD4$^+$ cells are labeled in red. (D) Visualization of the magnitude of infection (white arrows) 48 h following injection of Alexa Fluor-labeled AAV8 (Di) or unlabeled virus (Dii). (E) Visualization of the magnitude of infection 48 h following injection of Alexa Fluor-labeled AAV8 (Ei) and virus that underwent the same labeling procedure but did not include a fluorophore linked to the viral capsid (Eii). (F) Quantification of liver infection 48 h following treatment with labeled or sham-labeled virus (n = 3 for each group). (G) Quantification of the number of eYFP$^+$ cells at various time points following infection with AAV8 (n = 3 for controls, 5–6 for all other groups). For each animal, five fields of view (FOVs) were imaged and averaged. Data are presented as means ± SEM. $^*p < 0.05$, $^{**}p < 0.01$, $^{***}p < 0.001$ as compared to control. n.s., not significant. Scale bars, 50 μm.

(eYFP) in a mouse model of infection during a period of 56 days. We show that the injection of this AAV vector results in infection of hepatocytes and elicits a moderate, but significant, activation of the innate immune system in the liver. This inflammatory response involves platelet aggregation, neutrophil extracellular trap (NET) formation, and the recruitment of monocytes, B cells, and T cells. The depletion of liver macrophages delays the observed immune response, suggesting that liver Kupffer cells are required to initiate the host defense. Moreover, the death of liver cells produced by this AAV is moderate and fails to result in a robust inflammatory response. Altogether, these data support the use of AAV8 as a vehicle for gene therapy.

RESULTS

"Capture" of virus in the liver

Using IVM, we observed the rapid association of labeled virions on the surface of Kupffer cells and liver sinusoidal endothelium following intravenous (i.v.) administration of 4×10^{12} copies (cp)/kg of AAV8 (Figure 1A; Video S1). By tracking this viral capture in real time, individual virions can be seen being captured and directly binding to the surface of both Kupffer cells and endothelial cells without the need of other immune cells to bind or deliver the viral particles. As this process was monitored over time, the viral particles were lost from the surface of the endothelium, and by 30 min post-infection a clear accumulation of viruses on Kupffer cells and hepatocytes was observed (Figure 1B; Video S2). Utilizing an AAV8 virus engineered to deliver an eYFP gene to infected cells, we were able to track infection in the living animal using IVM. Using this approach on day 2 post-infection, we observed infection of several hepatocytes per field of view (FOV) (Figure 1C). Interestingly, we never observed an infected Kupffer cell, despite the clear accumulation of virions on the surface of these liver macrophages. This suggests that these cells can bind virus, but either the virus fails to enter the cell or the innate, intracellular antiviral response in Kupffer cells is sufficient to prevent infection.

Importantly, we observed reduced infection rates in animals treated with labeled virus compared to unlabeled AAV8 (Figure 1D). This indicated that the labeling process either modified the biological activity of the virions (e.g., trafficking of virus, cellular entry) or significant viral titer was lost during the labeling procedure. To ensure that the fluorescent labeling of the virions did not influence their bio-distribution or impact their ability to infect hepatocytes, we compared labeled virus to virus that underwent the same labeling procedure (incubations and centrifugations) but did not receive the labeling fluorophore. Comparing labeled virus to these sham-labeled virus, we observed infection of the same host cell type in the liver (hepatocytes) (Figure 1E) and found no difference in the number of infected cells following i.v. administration of virus (Figure 1F). Tracking infection over time, eYFP$^+$ hepatocytes begin to appear 1 day post-infection, with the number of infected cells increasing until approximately 5% (approximately 200 hepatocytes and 10 infected cells visible per FOV) of hepatocytes express eYFP by day 14 post-infection (Figure 1G). From day 14 until at least day 56, the number of infected hepatocytes appears relatively stable, demonstrating the ability of this AAV8 construct to generate a long-lived, chronic infection within a

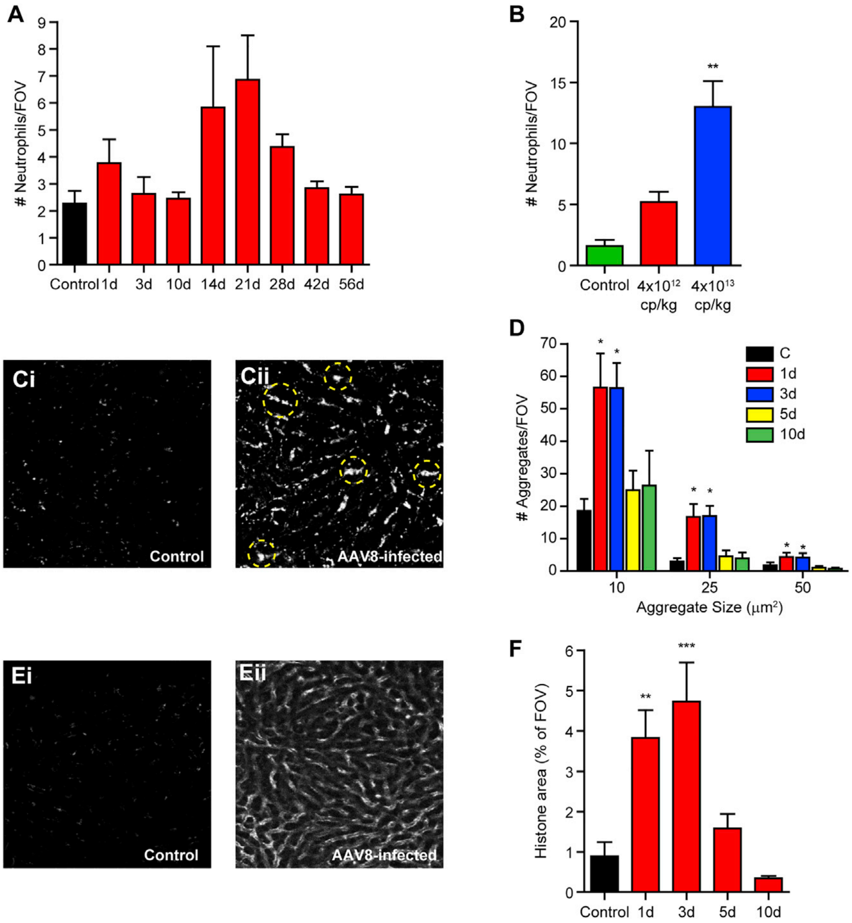

Figure 2. Acute inflammatory response following AAV8 infection

(A) Quantification of the number of neutrophils (Ly6G$^+$ cells) by IVM within the liver at various times following AAV8 infection (n = 3 for control, 5–6 for all other groups). (B) Quantification of neutrophil recruitment to the liver 48 h following challenge with two different infectious doses of AAV8 (n = 5 for each). (C) Representative IVM images of platelet aggregation in control (Ci) and AAV8-infected (Cii) liver 3 days post-infection. Large platelet aggregates are highlighted within yellow circles. (D) Quantification of platelet aggregates of indicated sizes at various time points following infection (n = 3 for controls, 5–6 for all other groups). (E) Representative IVM images of extracellular histone (NETs) in control (Ei) and AAV8-infected (Eii) liver 3 days post-infection. (F) Quantification of extracellular histone at various time points following infection (n = 5–6 for all groups). For each animal, five FOVs were imaged and averaged. Data are presented as means ± SEM. *$p < 0.05$, **$p < 0.01$, ***$p < 0.001$ as compared to control.

mouse model. With these experiments, we have validated the use of IVM to track AAV8-mediated infection of the mouse liver.

AAV8 induces acute inflammation, platelet aggregation, and NET formation

The early host response to most pathogens involves engagement of the innate immune system and often involves the recruitment of neutrophils to the site of infection. Previous work using live myxoma virus and the double-stranded RNA viral analog poly(I:C) had demonstrated that viral infection of the liver resulted in a rapid, robust cellular recruitment within the first day post-infection.[16] Tracking of neutrophil recruitment to the AAV8-infected liver fails to show a significant accumulation of cells at any time point (Figure 2A). This observation was surprising given previous studies utilizing other viruses and attests to the ability of AAV8 to infect without a strong host inflammatory response. Importantly, if the infectious dose of AAV8 is increased, neutrophil recruitment can be induced, suggesting that the virus is not completely immunologically "silent" (Figure 2B).

Recent studies in the liver have demonstrated significant platelet aggregation in response to inflammatory stimuli.[16–18] To see if AAV8 infection resulted in platelet activation and aggregation, we used IVM to track platelet dynamics in the liver in the first days post-infection. We noted significant platelet aggregation 1 and 3 days post-infection (4×10^{12} cp/kg) with the appearance of both small (10 μm^2) and large (>50 μm^2) aggregates within the liver vasculature (Figures 2C and 2D). These dynamic structures are continually building and sloughing off re-entering the circulation and do not appear to obstruct blood flow through this tissue (Video S3), suggesting that these structures are part of the host inflammatory response and do not represent true coagulation. Previous studies have demonstrated that this dynamic platelet aggregation is associated with the release of NETs.[19] NETs are immune effector mechanisms that are comprised of decondensed nuclear DNA released from activated neutrophils and decorated with both nuclear (histone) and granular proteins (e.g., myeloperoxidase [MPO], neutrophil elastase). These structures are designed to trap and sequester pathogens, limiting dissemination, but they have also been shown to kill bacteria directly.[20] This bactericidal activity is due to the fact these extracellular DNA structures are highly cytotoxic and can inflict significant collateral damage on host tissues. To determine whether the observed platelet activation leads to NET release following AAV8 administration, we stained for extracellular histone and looked into the liver by IVM in the first days following infection. Examination of the liver vasculature revealed a classic staining pattern of extracellular histone lining the sinusoidal walls, a pattern that is indicative of NET release (Figure 2E). Quantification of these structures showed significant NETs 1 and 3 days post-infection, with staining returning back to control levels by 5 days post-infection (Figure 2F).

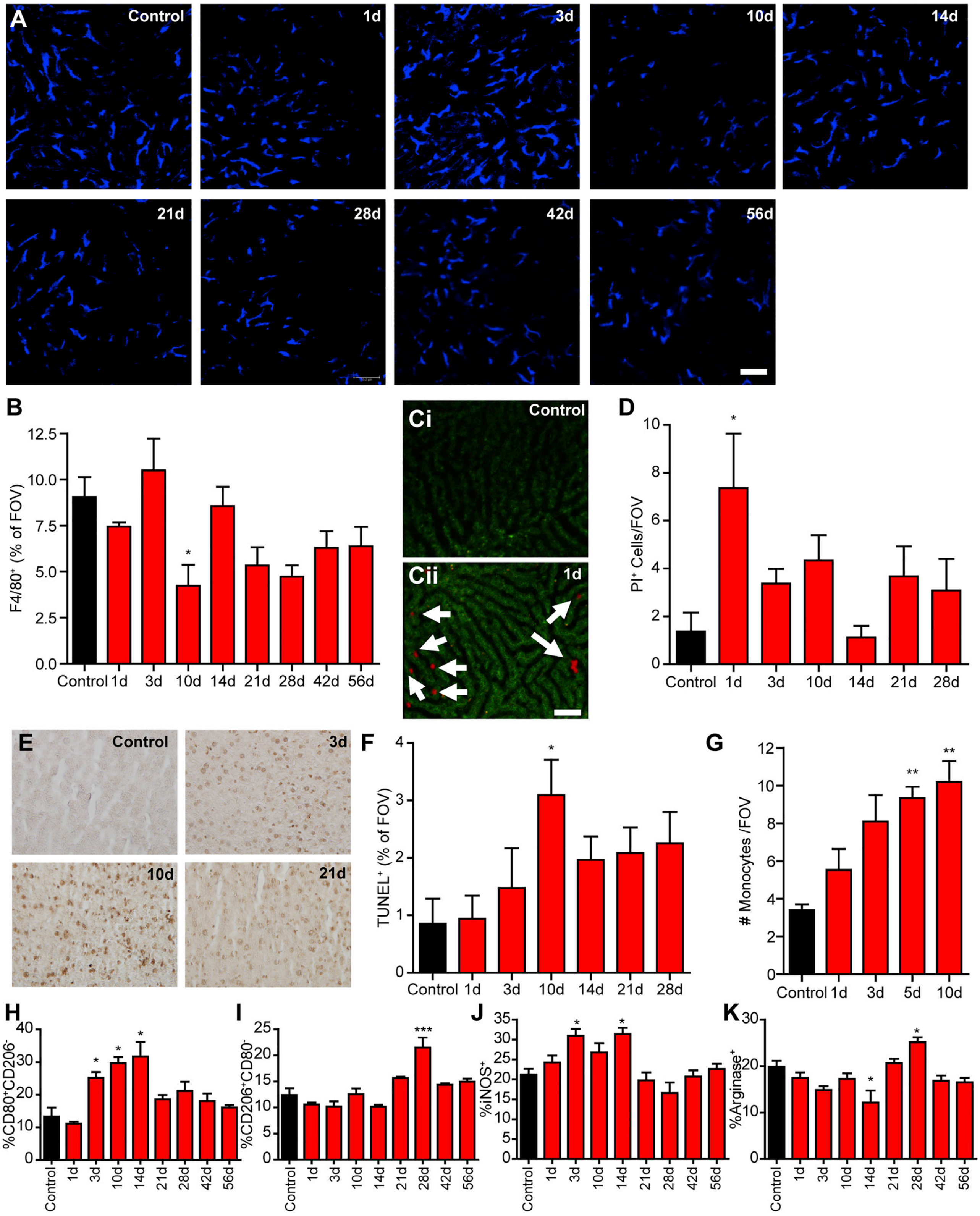

(legend on next page)

AAV8 infection results in loss of Kupffer cells and an increase in monocytes in the liver

Infection of the liver with the AAV8 viral vector results in an acute loss of resident liver macrophages, the Kupffer cells (Figure 3A). By 10 days post-infection this loss of F4/80$^+$ cells significant and slowly rebounds during the coming weeks (Figure 3B). Interestingly, this cell loss is associated with early markers of cell death. One day following infection, a significant increase in propidium iodide cells was observed, indicating the presence of lytic/necrotic cells in the liver (Figures 3C and 3D; Video S4). Following this acute phase of necrotic cell death, an increase in apoptotic cell death was observed (Figure 3E), peaking at approximately day 10 post-infection (Figure 3F). This loss of liver macrophage is paralleled by an increased recruitment of peripheral blood monocytes (CD11b$^+$Ly6G$^-$) to the liver vasculature (Figure 3G). Although classically associated with an inflammatory response, these monocytes begin to differentiate within the liver, reprogramming the liver microenvironment to a more anti-inflammatory state. Immediately following infection, monocyte and macrophage populations in the liver have a classic inflammatory M1 phenotype (CD80$^+$CD206$^-$; inducible nitric oxide synthase [iNOS]$^+$), but these inflammatory cells begin to transition to anti-inflammatory, reparative M2-like phenotype (CD80$^-$CD206$^+$; arginase$^+$) 2–3 weeks post-infection (Figures 3H–3K; Figure S1). This observation suggests that AAV8 infection reprograms the liver microenvironment to become less inflammatory, potentially limiting liver damage and prolonging infection.

Recruitment of adaptive immune cells

The immune response against the vector and/or the transgene product might preclude the expected therapeutic effect. Normally, efficient viral clearance requires engagement of the adaptive immune response, as specific and long-lasting antigen-specific immune responses are mediated by B and T cells. This adaptive host immune response involves the generation of neutralizing antibodies and CD8$^+$ cytotoxic T lymphocytes, regulated by the recruitment of helper and regulatory CD4$^+$ T cells.[21–25] To assay the ability of AAV8 infection to engage the adaptive immune response, we utilized IVM to track B and T cell recruitment to the liver after infection. Using multicolor fluorescence microscopy (Figure 4A) we were able to measure recruitment of B cells (CD19$^+$; Figure 4B), CD4$^+$ cells (Figure 4C), and CD8$^+$ T cells (Figure 4D) to the liver following AAV8 infection. With this approach, we observed a short-lived acute CD4$^+$ cell recruitment immediately following viral infection followed by later, sustained CD8$^+$ cell recruitment (out to at least 28 days post-infection). Because they are antigen-presenting cells, Kupffer cells may exert a significant influence on the expansion and activation, or tolerization, of antigen-specific CD4 and/or CD8 T cell-mediated immunity in the liver. Tracking cell behavior in infected mice, increasing percentages of CD8$^+$ T cells, and to a lesser extent CD4$^+$ cells, were observed making stable interactions with F4/80$^+$ liver macrophages in comparison to those occurring in non-infected control mice (Figures 4E–4G; Video S5).

Cytokine and chemokine responses to AAV8 infection

Infection, and subsequent cell death secondary to the infection, can trigger the production of several soluble inflammatory mediators that drive cellular recruitment and activation. Additionally, this cytokine milieu can contribute to the programing of the local tissue microenvironment, either driving inflammation or polarizing the tissue toward a more anti-inflammatory response. To understand this aspect of the host response to AAV8, we analyzed the concentration of cytokines and chemokines in plasma using a bead-based multiplex approach at various time points following viral infection. Interestingly, this cytokine response was very much muted following AAV8 infection, with no significant difference in the plasma concentration of most cytokines (granulocyte colony-stimulating factor [G-CSF], granulocyte-macrophage colony-stimulating factor [GM-CSF], interferon [INF]-γ, interleukin [IL]-1α, IL-1β, IL-2, IL-4, IL-3, IL-12 [p40 and p70], macrophage colony-stimulating factor [MCSF], MCP-1, MIP-1α, MIP-1β, MIP-2, RANTES, tumor necrosis factor [TNF]-α) (Figure 5). Although blunted overall, there was an early significant increase in IP-10, a chemoattractant for monocytes, T cells, and NK cells, and trends, albeit not significant, for elevated IL-6, IL-17, and KC in the early time points (days 1–3) post-infection. These cytokines and chemokines suggest the presence of an early inflammatory response in the liver following AAV8 infection. Additionally, there was a strong trend to increased IL-10 (anti-inflammatory) shortly after infection, and a significant increase in IL-7 (drives lymphocyte development) late in infection. These findings suggest a dynamic early inflammatory response that is rapidly curtailed, allowing for persistent AAV8 infection, and appear to align with leukocyte recruitment patterns observed in Figure 4.

Macrophage depletion prior to infection

Considering that macrophages are critical players in the immune response to infectious challenge by filtering the blood of pathogens,

Figure 3. Reprogramming of the liver microenvironment following AAV8 infection
(A) Representative images obtained by IVM of liver macrophages (blue) at various time points after infection. (B) Quantification of the area of F4/80 staining expressed as a percentage of FOV (n = 3 for control, 5–7 for all other groups). (C) Representative images obtained by IVM of dead/damaged cells in a control liver (Ci) and at 1 day post-infection (Cii) (propidium iodide [PI]$^+$, red; denoted by arrows) 24 h post-infection. (D) Quantification of the number of PI$^+$ cells per FOV at various time points post-infection (n = 3 for control, 5–6 for all other groups). (E) Representative images of TUNEL-stained liver sections obtained from mice at various time points post-infection. (F) Quantification of the area of TUNEL$^+$ staining per FOV at various time points post-infection (n = 3 for control, 5–6 for all other groups). (G) Quantification by IVM of the number of monocytes (CD11b$^+$Ly6G$^-$) per FOV at various time points post-infection (n = 3 for control, 5–6 for all other groups). (H–K) Fluorescence-activated cell sorting (FACS) analysis of macrophage phenotypes, CD80$^+$CD206$^-$ (H), CD80$^-$CD206$^+$ (I), iNOS$^+$ (J), and arginase$^+$ (K) cells, in the liver at various time points post-infection. All cells were pre-gated on size and F4/80-positive staining. Values represent the percentage of the liver macrophage population represented by each phenotype (n = 3 for control, 5–6 for all other groups). For each animal, five FOVs were imaged and averaged. Scale bars, 50 μm. Data are presented as means ± SEM. *p < 0.05, **p < 0.01, ***p < 0.001 as compared to control.

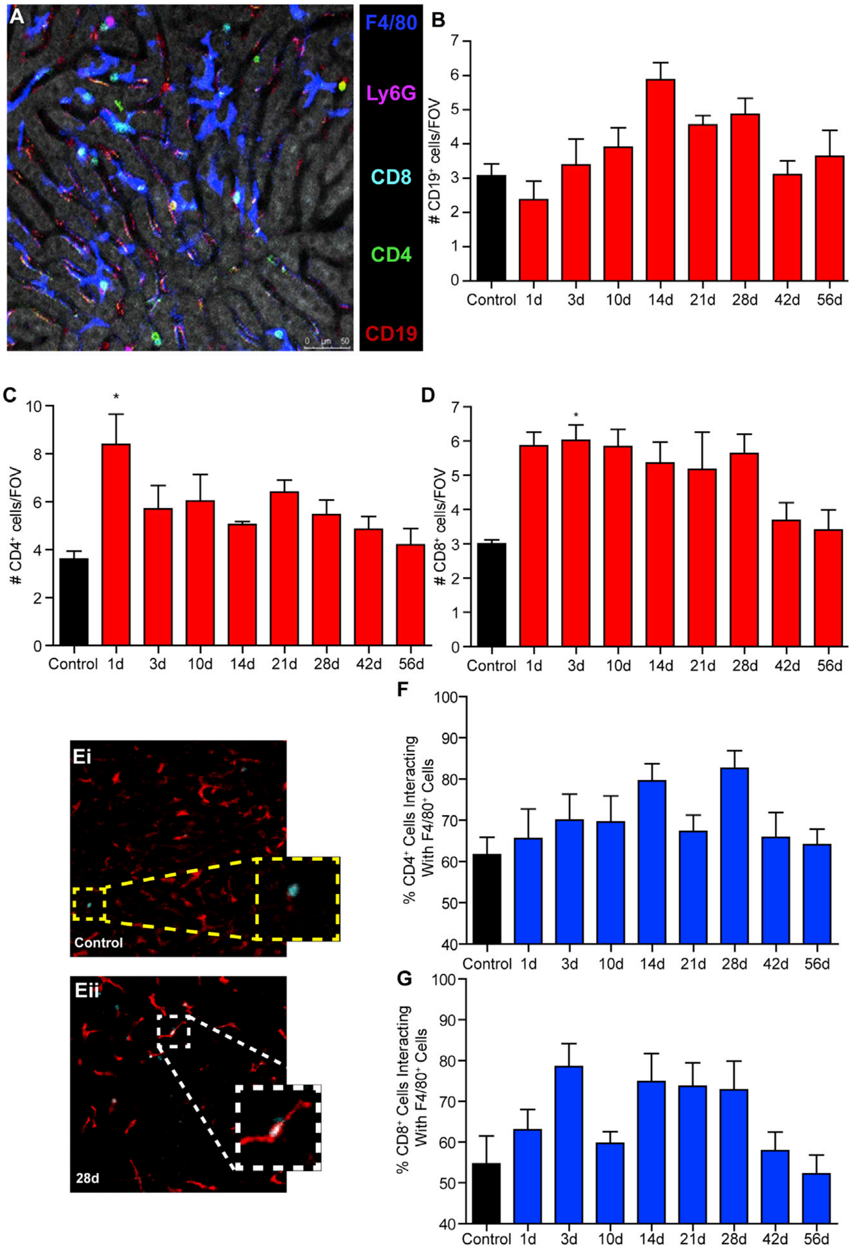

Figure 4. Lymphocyte recruitment to, and behavior within, the liver following AAV8 infection

(A) Representative image of multicolor IVM that allows for simultaneous quantification and tracking of multiple cell types in the live liver. Macrophages are shown in blue, neutrophils in purple, $CD8^+$ cells in cyan, $CD4^+$ cells in green, and B cells are shown in red. (B–D) Quantification of $CD19^+$ cells (B), $CD4^+$ cells (C), and $CD8^+$ cells (D) per FOV at various time points post-infection (n = 3 for control, 5–7 for all other groups). (E) Representative images from IVM showing $CD8^+$ cell-macrophage interaction within the liver of a control animal (Ei) and an animal at 28 days post-infection (Eii). $CD8^+$ cells not in contact with macrophages are denoted with a yellow-highlighted box, and cells in stable (>5 min) contact with macrophages are denoted with a white-highlighted box. (F and G) Quantification of $CD4^+$ (F) and $CD8^+$ cells (G) making stable contact with liver macrophages at various time points post-infection (n = 3 for control, 5–7 for all other groups). For each animal, five FOVs were imaged and averaged. Scale bar, 50 μm. Data are presented as means ± SEM. *$p < 0.05$ as compared to control.

initiating the innate immune response, and shaping the ensuing adaptive immunity, we sought to address the role that liver Kupffer cells play in AAV8 infection. Treatment of mice with clodronate liposomes (CLLs) i.v. 36 h prior to infectious challenge has been shown to deplete liver Kupffer cells (Figure 6A) while allowing peripheral blood monocytes to return to normal levels.[26,27] Following CLL treatment, the liver is slowly repopulated with macrophages, achieving levels comparable to non-CLL-treated AAV8-infected mice 2–3 weeks post-infection.

Although Kupffer cells bind a significant portion of i.v. delivered virus, depletion of liver macrophage did not increase the number of infected hepatocytes (Figure 6B); both the kinetics and the magnitude of hepatocyte infection remain unaltered in CLL-treated mice. Interestingly, despite no change in the magnitude of infection in CLL-treated mice, there was a significant impact on the overall immune response. Depletion of Kupffer cells resulted in attenuated neutrophil and monocyte recruitment following infection (Figures 6C and 6D). These data are particularly striking given that CLL treatment in non-infected mice resulted in elevated monocyte recruitment to the liver. Additionally, platelet aggregation, both large (>50 μm^2) and small (10 μm^2) aggregates, is significantly reduced in response to AAV8 infection of CLL-treated mice (Figure 6E). In agreement with reduced platelet aggregation, NET production following AAV8 infection was significantly impaired in CLL-treated animals (Figure 6F). CLL treatment resulted in a trend toward increased $CD4^+$ cell number within the liver, a pattern that remained constant throughout the duration of the assessment period (Figure 6G). In contrast, CLL treatment significantly delayed $CD8^+$ accumulation in the liver (Figure 6H), only reaching the number of $CD8^+$ cells observed in non-CLL-treated infected animals more than a week after AAV8 administration.

T cell depletion prior to infection

We next examined the role of T cells in AAV8 infection. Mice were depleted of $CD8^+$ or $CD4^+$ cells by injection of either an anti-CD8 (clone 2.43, Bio X Cell) or an anti-CD4 (clone GK1.5, Bio X Cell) monoclonal antibody, respectively.

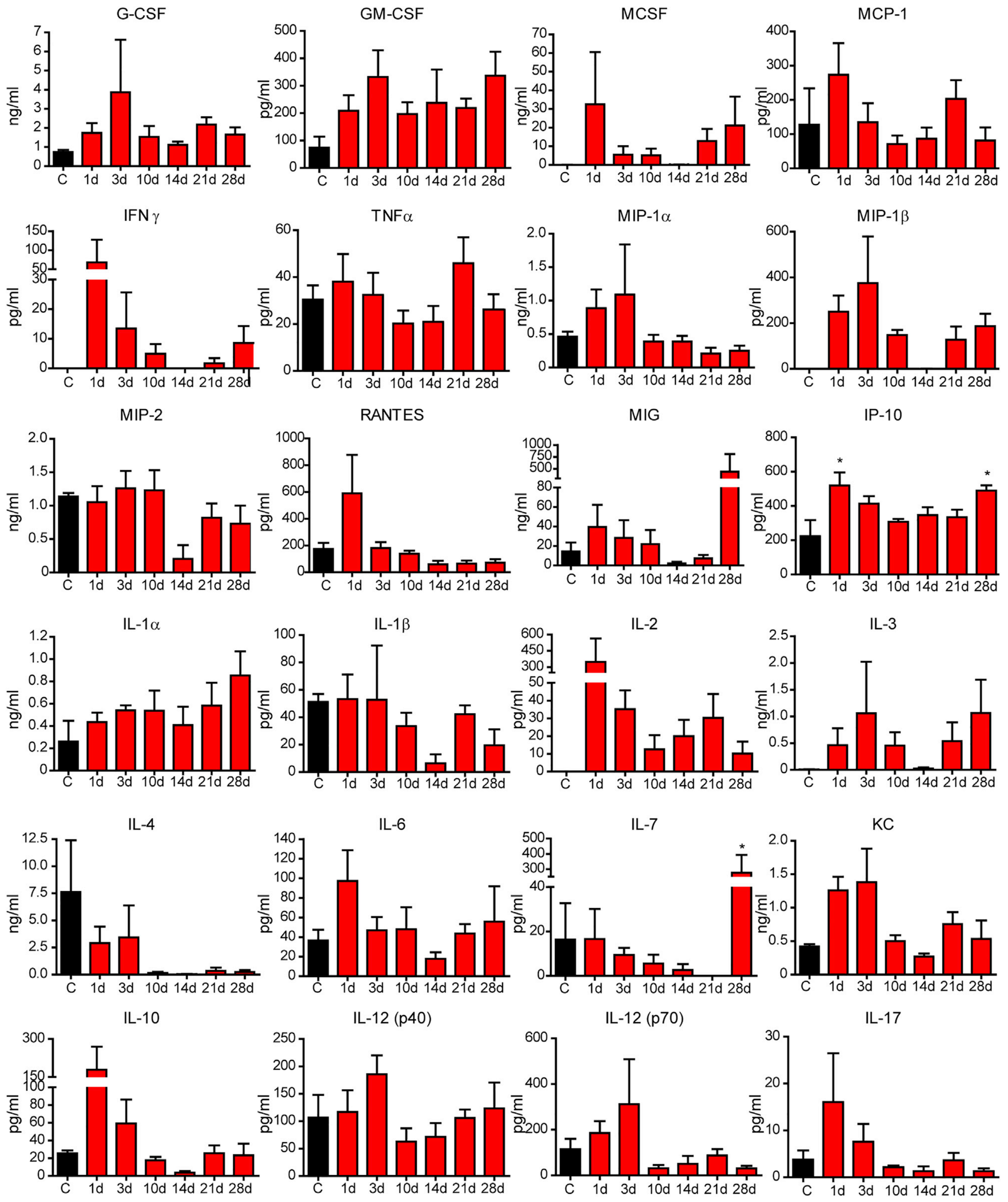

Figure 5. Systemic cytokine and chemokine levels at various time points following AAV8 infection

Plasma samples collected from mice at various time points post-infection were analyzed by bead-based multiplex quantification. All samples were run in duplicate and the mean value was used (n = 3 for control, 5–7 for all other groups). Data are presented as means ± SEM. *p < 0.05 as compared to control.

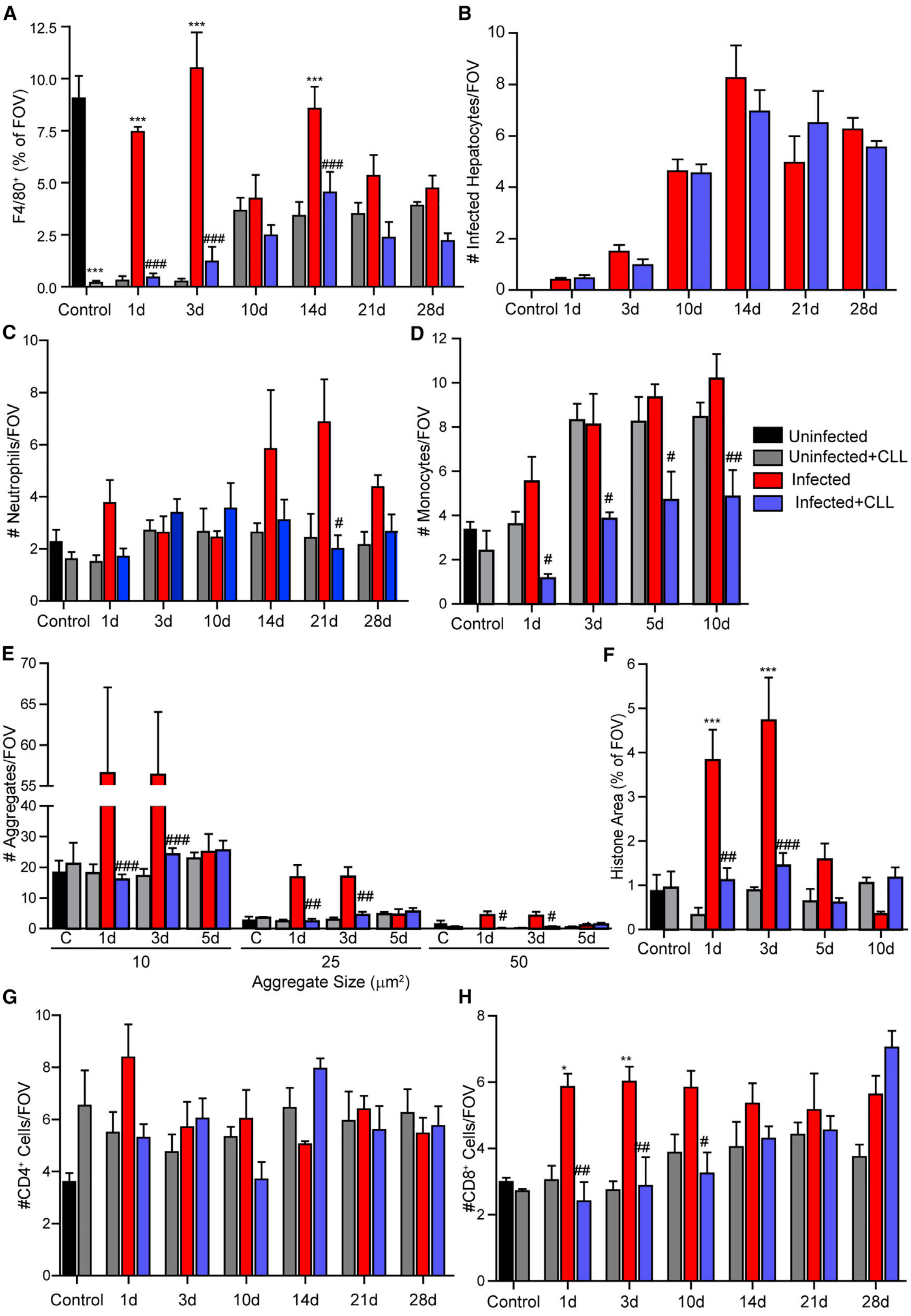

(legend on next page)

Interestingly, while CD8+ cell depletion resulted in reduced B cell (CD19+) recruitment to the liver (Figure 7E), there was no significant impact on the number of infected hepatocytes (Figure 7A), F4/80+ cells, CD4+ cell recruitment, or neutrophil cell numbers (Figures 7B–7D). In contrast, depletion of CD4+ cells significantly reduced the observed number of infected hepatocytes (Figure 7F). Depletion of CD4+ cells also resulted in a significant reduction of CD19+ cells in the liver but had little impact on F4/80+ cells, neutrophils, or CD8+ cell numbers (Figures 7G–7J).

DISCUSSION

Gene therapy offers promise for the treatment of heritable diseases that result from a dysfunctional version of protein.[4] By providing these patients a "working" copy of the defective gene, it is possible to cure some of these diseases, free patients from costly, ongoing therapies, and potentially save lives for patients afflicted with genetic illnesses for which there are no current therapies. The success of gene therapy is directly dependent of delivering the therapeutic genetic material to the patient in such a way that it (1) reaches the target tissues, (2) is transcribed over the long term, and (3) does not elicit a robust immune response that eliminates the "corrected" cells. Much effort has been focused on using gene therapy to treat blood disorders such as hemophilia.[28–30] This attention on blood disorders is largely due to the ability to use viral vectors to deliver therapeutic gene constructs to the liver, an organ that is able to secrete functional proteins directly into the blood and a tissue that is more immunologically tolerant than most. The microenvironment in the liver prevents robust activation of the adaptive immune system, a phenomenon that is exploited by some pathogens such as hepatitis B and hepatitis C viruses to establish chronic infection of the host.[31] This same immunotolerogenic niche can be used as an advantage for gene therapy, allowing for long-lived transduction of human cells, providing a durable, ongoing "cure" for these patients.

Delivery of the therapeutic DNA to the patient's cells can occur through a number of different mechanisms. In some cases, a patient's cells are cultured *in vitro* and are transfected with plasmid DNA to deliver the functional gene.[32] Following successful transfection, these cells are returned to the patient where they begin to express the transgene to produce therapeutic proteins. Another strategy is to use viruses to deliver the therapeutic DNA constructs directly into host cells.[33] The key features of a successful gene therapy vector are a high capacity of packaging size of the expression cassette and its easy purification into high titers to mediate targeted gene delivery and its prolonged gene expression with minimal immune response.[33] A number of different viruses have been utilized for this approach, including adenoviruses,[34] AAV,[7] retroviruses,[35] and lentiviruses.[36,37] Among these vectors, AAV8 has emerged as a strong platform candidate for the long-term delivery of gene constructs to the patient's liver, specifically transducing the hepatocytes, a very immunosuppressive cell type capable of inducing long-lived immune tolerance.[31] To better understand the host immune response to infection by AAV8 we utilized IVM to look directly into the liver of live mice to visualize viral delivery and to assess engagement of the immune system at various times post-infection.

Upon i.v. delivery of AAV8, virus can be seen associating with and accumulating on the surface of both liver macrophage Kupffer cells and vascular endothelial cells (Figure 1A). Importantly, although both cell types appear able to capture virus from the bloodstream, neither appears to be infected by the virus, as demonstrated by a lack of eYFP expression in either cell type. In contrast, following the initial bolus of virus (30 min post-injection), individual virions can be observed accumulating on the surface of these cells (Figure 1B). It is these hepatocytes that become infected and express the viral gene payload, eYFP (Figure 1G). This viral gene expression begins to become apparent 24 h post-infection with the appearance of the first eYFP+ cells. During the next several days, additional cells begin expressing the viral payload, reaching a maximal number of eYFP+ cells at 14 days post-infection. The expression of these viral payloads continues for at least 56 days, indicating that this approach is able to provide a long-term solution to expression of a delivered gene.

Importantly, although Kupffer cells do not appear to become infected by AAV8, the interaction of virus with these cells is not immunologically silent. Following viral delivery there is a rapid recruitment of platelets (Figure 2D) to the liver. Although this response is rather mild when compared to what has been previously reported for other viral infections,[16] it does result in the release of NETs from neutrophils within the liver vasculature (Figure 2F), structures that have been shown to drive the host inflammatory response leading to vascular dysfunction and tissue damage.[17] Importantly, this inflammatory response is short-lived, with resolution of platelet aggregation and NETs as early as 5 days post-infection, despite the ongoing expression of the viral payload within the hepatocytes.

Figure 6. Effect of Kupffer cell depletion on AAV8 infection and the ensuing immune response

(A) Quantification of liver macrophages by IVM in control, CLL-treated, and AAV8-infected animals at various time points post-infection. Values represent the area of F4/80 staining as a percentage of a FOV (n = 2 for control, 4–6 for all other groups). (B) Number of infected (eYFP+) cells at various time points following infection in wild-type and CLL-treated mice (n = 3 for controls, 5–10 for all other groups). (C) Number of neutrophils within the liver at various time points following infection in wild-type and CLL-treated mice (n = 3 for controls, 5–10 for all other groups). (D) Number of monocytes within the liver at various time points following infection in wild-type and CLL-treated mice (n = 3 for controls, 5–10 for all other groups). (E) Quantification of platelet aggregates of indicated sizes at various time points following infection in control and CLL-treated animals (n = 3 for controls, 5–10 for all other groups). (F) Quantification of extracellular histone at various time points following infection in control and CLL-treated animals (n = 3 for controls, 5–10 for all other groups). (G and H) Number of CD4+ (G) and CD8+ (H) cells in the liver at various time points following infection with AAV8 in control and CLL-treated animals (n = 3 for controls, 4–6 for all other groups). For each animal, five FOVs were imaged and averaged. Data are presented as means ± SEM. *p < 0.05, **p < 0.01, ***p < 0.001 as compared to uninfected-CLL treated animal at each time point; #p < 0.05, ##p < 0.01, ###p < 0.001 as compared to infected animal at each time point.

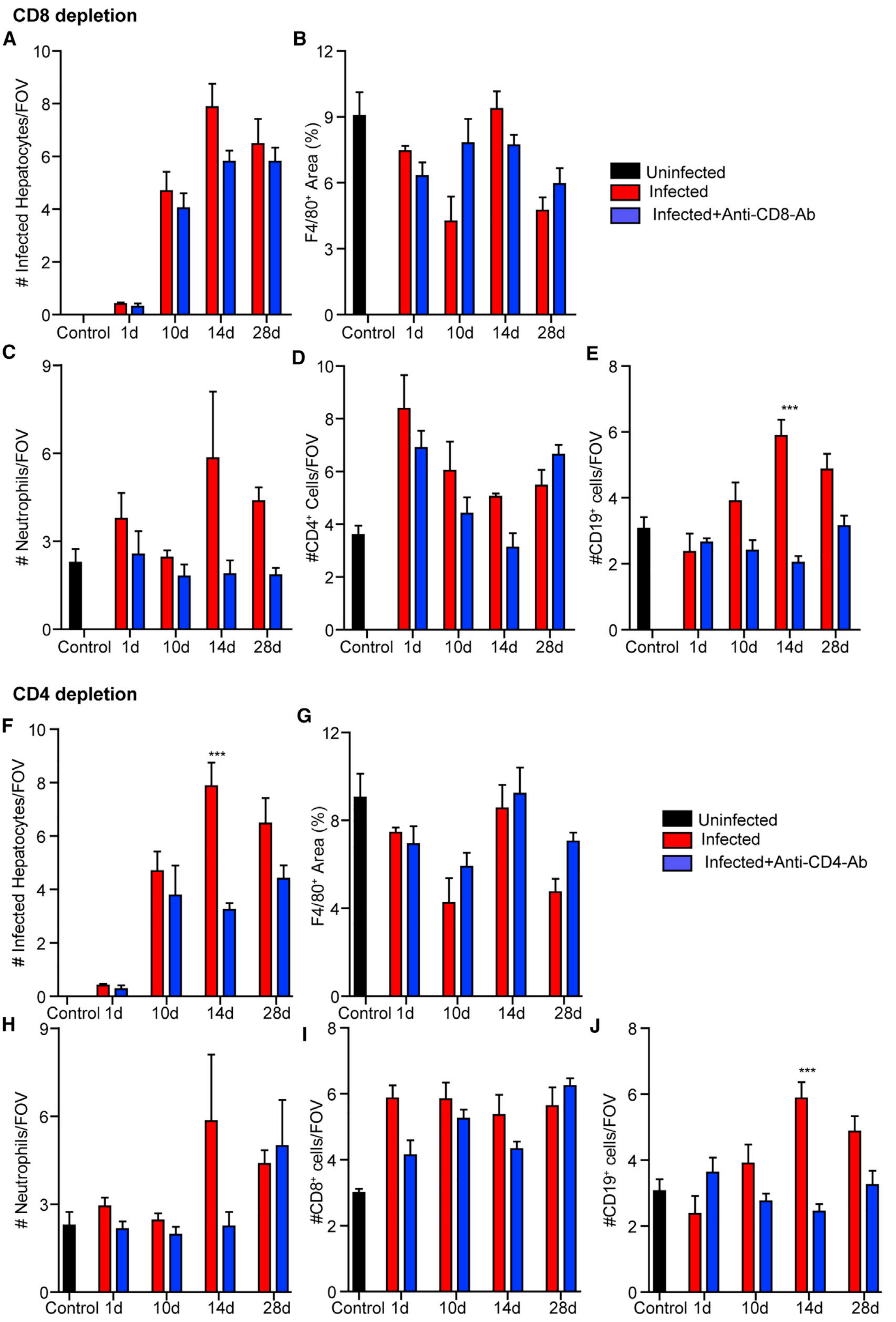

(legend on next page)

In addition to neutrophils, platelets, and NETs, AAV8 infection directly impacts the liver macrophage population itself. Following AAV8 infection, a significant loss of Kupffer cells is observed (Figure 3B). This macrophage loss is associated with the observation of markers of both cell necrosis (propidium iodide; Figure 3D) and apoptosis (TUNEL [terminal deoxynucleotidyltransferase-mediated deoxyuridine triphosphate nick end labeling assay]; Figure 3F). This loss of liver macrophage is paralleled by the accumulation of peripheral blood monocytes within the liver (Figure 3G). Moreover, as these recruited cells begin to differentiate into macrophages, they reprogram the liver from an overall pro-inflammatory phenotype into an anti-inflammatory microenvironment (Figures 3H–3K). This switch from inflammatory to anti-inflammatory corresponds directly to the accumulation of $CD8^+$ T cells in the liver and the interaction between these cells and the M2-like anti-inflammatory macrophages (Figures 4D and 4G), suggesting a mechanism of tolerance to the viral infection. Interestingly, despite this clear reprogramming of the liver microenvironment, a systemic cytokine response was not observed (Figure 5). This indicates immune reprograming is a local event and that AAV8 infection fails to elicit a systemic inflammatory response. This relative immunological "silence" may help explain how AAV8 is able to establish a long-lived infection within the liver.

Importantly, the activation of the host immune response (cell recruitment, platelet aggregation, NET release) is directly coordinated by the liver macrophage. Depletion of Kupffer cells prior to infection attenuates all observed immune responses, limiting inflammation and delaying lymphocyte recruitment to the liver (Figures 6C–6H). Although Kupffer cells appear to be an important clearance mechanism for i.v. delivered virus, including binding and sequestering substantial quantities of virus, depletion of these cells surprisingly did not increase the overall infection of the liver, indicating that the resident macrophage population is not a barrier to gene delivery to the hepatocytes (Figure 6B).

Interesting results were obtained in animals depleted of specific T cell populations. Given the central role of cytotoxic T cells in the resolution of viral infection, it was surprising that depletion of $CD8^+$ T cells failed to impact the number of virally infected hepatocytes observed through the duration of the experiment (28 days post-infection) (Figure 7A). In contrast, depletion of $CD4^+$ T cells resulted in a loss of virally infected hepatocytes observed 2 weeks post-infection (Figure 7F). Although it is unclear how depletion of $CD4^+$ cells may have resulted in enhanced viral clearance, it is noteworthy that the depleting antibody used was not only specific to the Th1 and Th2 cells subsets, but it also eliminates other $CD4^+$ cell populations. This is particularly important if one considers that the liver contains a substantial population of resident regulatory T cells (Tregs),[38] cells that once deleted could potentially relax the breaks on the host immune response within the liver. Interestingly, depletion of either $CD8^+$ (Figure 7E) or $CD4^+$ cells (Figure 7J) resulted in a transient increase in B lymphocytes within the liver 14 days post-infection. This increased recruitment of B cells may represent a compensatory response to viral infection in the absence of functional T cell immunity, although the specific pathways involved require additional study before we can fully understand the potential interplay between B and T cells within the infected liver.

The use of IVM to directly observe and characterize the host immune response to AAV8 infection has provided great insight into the interactions between this gene therapy vector and the immune system. These studies specifically address the use of AAV8 as a gene therapy platform and begin to map the basic immune response to this vector during the first several weeks of infection. It will be particularly interesting in future studies to determine the impact of other viral strains and vector systems on observed initial immune response, comparing any potential differences to the AAV8 baseline response observed in this study. We have shown that the liver-resident macrophage population, the Kupffer cells, are the principal immune sentinels in the liver, alerting the immune system to the presence of viral infection. Although Kupffer cells do not limit viral delivery to hepatocytes, they do activate and coordinate the early, acute inflammatory response and the later recruitment of lymphocytes to the infected liver. This places the Kupffer cell as a potential target for immunomodulation as a co-therapy to be used along with virus-based gene therapy. Preventing the activation and recruitment of neutrophils and platelets, the release of NETs and the associated tissue damage will improve overall patient outcomes and may reduce the overall immune response to the vector-infected host cells.

Additionally, we have demonstrated that the sustained infection mediated by AAV8 involves reprograming of the liver microenvironment, shifting the macrophage population from a pro-inflammatory M1-like phenotype toward an M2-like, anti-inflammatory/reparative phenotype. It is important to determine whether this shift can be enhanced through the administration of exogenous cytokines, elevating liver infection and helping to ensure the long-term survival of transduced cells.

Figure 7. Effect of depletion of specific T cell populations on AAV8 infection and the ensuing immune response

(A–J) Mice were treated with a monoclonal antibody to specifically deplete either $CD8^+$ (A–E) or $CD4^+$ (F–J) cells throughout the duration of the infection. (A and F) Numbers of infected ($eYFP^+$) cells at various time points following infection in control, anti-CD8-Ab-treated (A), or anti-CD4-Ab-treated (F) mice (n = 3 for controls, 5 for all other groups). (B and G) Quantification of liver macrophages following AAV8 infection by IVM in control, anti-CD8-Ab-treated (B), or anti-CD4-Ab-treated (G) mice at various time points post-infection. Values represent the area of F4/80 staining as a percentage of a FOV (n = 3 for control, 5 for all other groups). (C and H) Number of neutrophils within the liver at various time points following infection in control, anti-CD8-Ab-treated (C), or anti-CD4-Ab-treated (H) mice (n = 3 for controls, 5 for all other groups). (D and E) Number of $CD4^+$ (D) and $CD19^+$ cells (E) within the liver at various time points following infection in control and anti-CD8-Ab-treated mice (n = 3 for controls, 5 for all other groups). (I and J) Number of $CD8^+$ (I) and $CD19^+$ cells (J) within the liver at various time points following infection in control and anti-CD4-Ab-treated mice (n = 3 for controls, 5 for all other groups). For each animal, five FOVs were imaged and averaged. Data are presented as means ± SEM. ***$p < 0.001$ as compared to non-treated animal at each time point.

Understanding how viral gene therapy vectors engage the host immune response and reshape the liver microenvironment has helped identify novel therapeutic targets that may enhance/stabilize viral delivery of gene constructs. Future gene therapy strategies may aim to modulate this early immune response, preventing inflammation and limiting the generation of anti-viral immunity. This approach may help extend the therapeutic effect of gene therapy treatment, allowing patients to receive fewer treatments and to achieve long-term cures.

MATERIALS AND METHODS

AAV viral vector

One lot of AAV vector GT012 was used for all studies. The vector's single-stranded genome of 3,379 bp is packaged into an AAV8 capsid and harbors an eYFP expression cassette driven by the cytomegalovirus (CMV) immediate early enhancer/promoter, which is flanked by AAV2 inverted terminal repeats. The additional presence of an SV40 promoter-driven neomycin resistance gene served to increase the size of the cassette but was otherwise irrelevant for the present study.

The vector was produced using a triple-plasmid transfection protocol for HEK293 cells and purified from clarified cell lysates by sequential density gradient centrifugation and ion exchange chromatography according to Grieger et al.[39] The resulting lot of vector GT012 had a titer of 3.0×10^{13} cp/mL, determined by AAV8 titration ELISA (Progen, Heidelberg, Germany), and no detectable endotoxin activity (<0.5 endotoxin units [EU]/mL).

Mice

C57BL/6 mice were purchased from Jackson Laboratory (Bar Harbor, ME, USA). Mice used in this study were 6–8 weeks of age and were maintained in a specific pathogen-free environment at the University of Calgary Animal Resource Centre. All experimental animal protocols were approved by the University of Calgary Animal Care Committee (AC16-0218) and were in compliance with guidelines from the Canadian Council for Animal Care.

Infection and treatment of mice

Unless otherwise indicated, mice were infected with 4×10^{12} cp/kg of AAV8. The virus stock was diluted in PBS and 40 μL was injected i.v. and analyzed after 1, 3, 5, 10, 14, 21, 28, 42, and 56 days of infection. Control animals received 40 μL of PBS i.v. 24 h prior to imaging or tissue collection. In some experiments, mice were pre-treated with 200 μL of CLL (Liposoma, Amsterdam, the Netherlands) intraperitoneally (i.p.), 36 h prior the infection to deplete liver macrophages. In other experiments, mice were injected i.p. with an initial dose of 200 μg of anti-CD8 (clone 2.43, Bio X Cell) or anti-CD4 (clone GK1.5, Bio X Cell) antibody, followed by a subsequent doses of 200 μg of depleting antibody every 7 days throughout the infection.

Antibodies

For IVM, Brilliant Violet 421-labeled anti-Ly6G (clone 1A8), phycoerythrin (PE)-labeled anti-CD49b (clone HMα2), peridinin chlorophyll protein (PerCP)-Cy5.5-labeled anti-CD11b (clone M1/70), PE-labeled anti-F4/80 (clone BM8), Alexa Fluor 647-labeled anti-CD4 (clone GK1.5), PerCP-Cy5.5-labeled anti-CD8 (clone 53-6.7), and allophycocyanin (APC)-R700-labeled anti-CD19 (clone 1D3) were purchased from BioLegend (San Diego, CA, USA). Goat anti-mouse histone H2Ax (clone M20, Santa Cruz Biotechnology, USA) was conjugated to Alexa Fluor 647 using a protein labeling kit as per the manufacturer's instructions (Life Technologies, Carlsbad, CA, USA). Typically, 1.5 μg of each Ab is injected i.v. to label the desired targets in live mice.

For flow cytometry, APC-Fire 750-labeled anti-F4/80 (clone BM8), PerCP-Cy5.5-labeled anti-CD80 (clone 16-10A1), and Alexa Fluor 647-labeled anti-CD206 (clone C068C2) were purchased from BioLegend (San Diego, CA, USA). PE-labeled anti-arginase I and PE-Cy7 anti-NOS2 (clone CXNFT) were purchased from R&D Systems (Minneapolis, MN, USA) and Thermo Fisher Scientific (Waltham, MA, USA), respectively.

Virus labeling

Alexa Fluor 647 succinimidyl esters (Molecular Probes, Invitrogen) were reconstituted in DMSO. Five-microliter serial dilutions (10, 3, and 1 mg/mL and 300, 200, 100, 30, 20, and 10 μg/mL prepared in PBS) were added to 45 μL of AAV for final concentrations of 1,000, 300, 100, 30, 20, 10, 3, 2, and 1 μg/mL dye, while stirring gently. Virus and dye were incubated 20, 40, or 120 min at room temperature, with gentle inversions every 5–15 min. Unbound dye was removed by transferring the labeling mixture to Amicon Ultra-4 centrifugal filter units (100-kDa membrane; EMD Millipore) and washing twice in 1 mL of PBS by centrifugation (4,000 × *g*, 10 min, 4°C). Control virus samples were incubated with PBS.

IVM

Mice were maintained under general anesthetic with a mixture of ketamine hydrochloride (200 mg/kg, Rogar/SBT) and xylazine hydrochloride (10 mg/kg, MTC Pharmaceuticals) for the duration of imaging experiments. After induction of anesthesia, the tail vein was cannulated for administration of additional anesthetic and injection of antibodies or other reagents. The liver preparation procedure was executed as previously described.[17] Briefly, following a midline incision of the skin, the exposed tissue was cauterized and then removed. The aforementioned procedure was repeated to the peritoneum, exposing the liver. The hepatic ligaments were dissected, and the sternum was secured with a suture. The mouse was transferred to a heated stage to maintain body temperature throughout imaging and positioned on the right side. The intestines were displaced and contained within a moistened gauze. The liver was positioned on a glass coverslip by maneuvering the stomach with a moistened cotton swab. To reduce liver movement during imaging and mimic physiological conditions, a moistened single-ply tissue was draped over the exposed liver. Imaging was performed on a resonant-scanning confocal microscope (TCS-SP8, Leica Microsystems, Concord, ON, Canada) equipped with 405-, 488-, 552-, and 638-nm excitation lasers, an 8-kHz tandem scan head, and spectral detectors (conventional photomultiplier

tube [PMT] and hybrid HyD detectors), using a ×25 water objective lens. Leica Application Suite X (Leica Microsystems) was used to control the microscope and record images.

Analysis of resonant-scanning confocal microscope-acquired images

Enumeration of neutrophils, imaging of NETs, platelet aggregates, and thrombin was conducted using IVM as previously described.[40] Snapshots were generated from intravital videos, and images were exported in a tif format. The same contrast and threshold values were applied to all images from all treatment groups within the experiment. For platelet aggregation and Kupffer cell quantification, thresholded images were converted to a binary (black and white) format, and the area per FOV covered by positive fluorescence staining (black) was calculated with ImageJ software. Data are expressed as the percentage of area in each FOV covered by positive fluorescence staining. Analysis of the total area of staining was done with ImageJ. A common minimum brightness threshold was set for all images to eliminate background autofluorescence, and the resulting images were converted to a binary format. Total area corresponding to fluorescence staining was measured for each FOV, and values are expressed as mm^2.

Analysis of macrophage polarization by flow cytometry

The liver was obtained from animals after different periods of AAV8 infection. A hepatocyte suspension was obtained by mechanical separation using a 60-μm mesh. After centrifugation, erythrocytes were lysed with hypotonic buffer and, after washing, the hepatocytes (5×10^6) were blocked and stained for F4/80, CD80, and CD206 for 30 min on ice. To determine intracellular iNOS and arginase, the cells were washed, fixed, and permeabilized with a Cytofix/Cytoperm kit (BD Bioscience). After 30 min of incubation with labeling Abs, the cells were washed and analyzed using an Attune NxT flow cytometer (Thermo Fisher Scientific).

Quantitation of cytokines

Blood was extracted at different time points after infection from mice. Plasma was obtained by centrifuging whole blood at 3,000 rpm for 10 min to measure 29 cytokines, including G-CSF, GM-CSF, IFN-γ, IL-1α, IL-1β, IL-2, IL-3, IL-4, IL-5, IL-6, IL-7, IL-10, IL-12 (p40), IL-12 (p70), IL-13, LIX, IL-15, IL-17, IP-10, KC, M-CSF, MCP-1, MIP-1α, MIP-1β, MIP-2, RANTES, TNF-α, MIG, and LIF by a Luminex bead-based multiplex array. The assay was performed according to the manufacturer's instructions (EMD Millipore, Merck).

TUNEL assay

Livers were collected, fixed in formalin, embedded in paraffin, and sectioned. Slides were deparaffinized in xylene three times for 5 min and rehydrated with descending concentrations of ethanol. A TUNEL assay was used to detect the DNA strain breaks. The tissue sections were stained using the Click-iT TUNEL colorimetric immunohistochemistry (IHC) detection kit (Thermo Fisher Scientific, USA) as per the manufacturer's instructions. Apoptotic and necrotic cells were stained with 1× 3,3′-diaminobenzidine (DAB) reaction mixture supplied by the kit. The apoptotic cell nuclei were stained in brown.

Statistical analysis

All results are presented as mean values ± SEM. The overall significance of the data was examined by one-way or two-way analysis of variance, with Tukey's post hoc test used for multiple comparisons. Differences between the groups were considered statistically significant at a $p < 0.05$.

SUPPLEMENTAL INFORMATION

Supplemental Information can be found online at https://doi.org/10.1016/j.omtm.2020.10.023.

ACKNOWLEDGMENTS

This work was supported by a research service grant from Baxalta Innovations GmbH, a member of the Takeda group of companies. A.C. was supported by the Beverley Phillips Rising Star Postdoctoral Fellowship and the University of Calgary, Cumming School of Medicine Postdoctoral Scholar Program. C.N.J. was supported by the Canada Research Chairs Program.

AUTHOR CONTRIBUTIONS

A.C. performed the experiments, analyzed the data, and wrote the manuscript. S.-J.K. performed experiments and analyzed the data. F.H. provided essential reagents, performed experiments, and reviewed and edited the manuscript. H.R. provided essential reagents and wrote, reviewed, and edited the manuscript. C.L. participated in study design, managed part of the study, reviewed and interpreted data, and reviewed and edited the manuscript. B.M.R generated the idea for the study, participated in study design, reviewed and interpreted data, and reviewed and edited the manuscript. B.A.C. managed part of the study, reviewed and interpreted data, and wrote, reviewed, and edited the manuscript. C.N.J. conceived, designed, and supervised the research, analyzed data, and wrote the manuscript.

DECLARATION OF INTERESTS

F.H., H.R., B.M.R., and B.A.C. were employees of Baxalta Innovations GmbH, a member of the Takeda group of companies, Vienna, Austria, at the time of the study. F.H., H.R. and B.A.C. are stockholders in Takeda Pharmaceutical. The remaining authors declare no competing interests.

REFERENCES

1. McCandless, S.E., Brunger, J.W., and Cassidy, S.B. (2004). The burden of genetic disease on inpatient care in a children's hospital. Am. J. Hum. Genet. *74*, 121–127.
2. Friedmann, T. (1992). A brief history of gene therapy. Nat. Genet. *2*, 93–98.
3. Misra, S. (2013). Human gene therapy: a brief overview of the genetic revolution. J. Assoc. Physicians India *61*, 127–133.
4. Mingozzi, F., and High, K.A. (2011). Therapeutic in vivo gene transfer for genetic disease using AAV: progress and challenges. Nat. Rev. Genet. *12*, 341–355.
5. Gao, G.-P., Alvira, M.R., Wang, L., Calcedo, R., Johnston, J., and Wilson, J.M. (2002). Novel adeno-associated viruses from rhesus monkeys as vectors for human gene therapy. Proc. Natl. Acad. Sci. USA *99*, 11854–11859.

6. Zinn, E., and Vandenberghe, L.H. (2014). Adeno-associated virus: fit to serve. Curr. Opin. Virol. *8*, 90–97.
7. Naso, M.F., Tomkowicz, B., Perry, W.L., 3rd, and Strohl, W.R. (2017). Adeno-associated virus (AAV) as a vector for gene therapy. BioDrugs *31*, 317–334.
8. Kaplitt, M.G., Feigin, A., Tang, C., Fitzsimons, H.L., Mattis, P., Lawlor, P.A., Bland, R.J., Young, D., Strybing, K., Eidelberg, D., and During, M.J. (2007). Safety and tolerability of gene therapy with an adeno-associated virus (AAV) borne GAD gene for Parkinson's disease: an open label, phase I trial. Lancet *369*, 2097–2105.
9. Vandendriessche, T., Thorrez, L., Acosta-Sanchez, A., Petrus, I., Wang, L., Ma, L., DE Waele, L., Iwasaki, Y., Gillijns, V., Wilson, J.M., et al. (2007). Efficacy and safety of adeno-associated viral vectors based on serotype 8 and 9 vs. lentiviral vectors for hemophilia B gene therapy. J. Thromb. Haemost. *5*, 16–24.
10. Zincarelli, C., Soltys, S., Rengo, G., and Rabinowitz, J.E. (2008). Analysis of AAV serotypes 1–9 mediated gene expression and tropism in mice after systemic injection. Mol. Ther. *16*, 1073–1080.
11. Grieger, J.C., and Samulski, R.J. (2005). Packaging capacity of adeno-associated virus serotypes: impact of larger genomes on infectivity and postentry steps. J. Virol. *79*, 9933–9944.
12. Wang, L., Wang, H., Bell, P., McMenamin, D., and Wilson, J.M. (2012). Hepatic gene transfer in neonatal mice by adeno-associated virus serotype 8 vector. Hum. Gene Ther. *23*, 533–539.
13. Jooss, K., and Chirmule, N. (2003). Immunity to adenovirus and adeno-associated viral vectors: implications for gene therapy. Gene Ther. *10*, 955–963.
14. Boutin, S., Monteilhet, V., Veron, P., Leborgne, C., Benveniste, O., Montus, M.F., and Masurier, C. (2010). Prevalence of serum IgG and neutralizing factors against adeno-associated virus (AAV) types 1, 2, 5, 6, 8, and 9 in the healthy population: implications for gene therapy using AAV vectors. Hum. Gene Ther. *21*, 704–712.
15. Calcedo, R., Vandenberghe, L.H., Gao, G., Lin, J., and Wilson, J.M. (2009). Worldwide epidemiology of neutralizing antibodies to adeno-associated viruses. J. Infect. Dis. *199*, 381–390.
16. Jenne, C.N., Wong, C.H.Y., Zemp, F.J., McDonald, B., Rahman, M.M., Forsyth, P.A., McFadden, G., and Kubes, P. (2013). Neutrophils recruited to sites of infection protect from virus challenge by releasing neutrophil extracellular traps. Cell Host Microbe *13*, 169–180.
17. McDonald, B., Davis, R.P., Kim, S.-J., Tse, M., Esmon, C.T., Kolaczkowska, E., and Jenne, C.N. (2017). Platelets and neutrophil extracellular traps collaborate to promote intravascular coagulation during sepsis in mice. Blood *129*, 1357–1367.
18. van Golen, R.F., Stevens, K.M., Colarusso, P., Jaeschke, H., and Heger, M. (2015). Platelet aggregation but not activation and degranulation during the acute post-ischemic reperfusion phase in livers with no underlying disease. J. Clin. Transl. Res. *1*, 107–115.
19. Clark, S.R., Ma, A.C., Tavener, S.A., McDonald, B., Goodarzi, Z., Kelly, M.M., Patel, K.D., Chakrabarti, S., McAvoy, E., Sinclair, G.D., et al. (2007). Platelet TLR4 activates neutrophil extracellular traps to ensnare bacteria in septic blood. Nat. Med. *13*, 463–469.
20. McDonald, B., Urrutia, R., Yipp, B.G., Jenne, C.N., and Kubes, P. (2012). Intravascular neutrophil extracellular traps capture bacteria from the bloodstream during sepsis. Cell Host Microbe *12*, 324–333.
21. Mingozzi, F., Maus, M.V., Hui, D.J., Sabatino, D.E., Murphy, S.L., Rasko, J.E.J., Ragni, M.V., Manno, C.S., Sommer, J., Jiang, H., et al. (2007). $CD8^+$ T-cell responses to adeno-associated virus capsid in humans. Nat. Med. *13*, 419–422.
22. Grubbs, H., and Kahwaji, C.I. (2018). Physiology, active immunity. In StatPearls, https://www.ncbi.nlm.nih.gov/pubmed/30020652.
23. Bessis, N., GarciaCozar, F.J., and Boissier, M.C. (2004). Immune responses to gene therapy vectors: influence on vector function and effector mechanisms. Gene Ther. *11* (*Suppl 1*), S10–S17.
24. Tang, J., Olive, M., Pulmanausahakul, R., Schnell, M., Flomenberg, N., Eisenlohr, L., and Flomenberg, P. (2006). Human $CD8^+$ cytotoxic T cell responses to adenovirus capsid proteins. Virology *350*, 312–322.
25. Calcedo, R., Chichester, J.A., and Wilson, J.M. (2018). Assessment of humoral, innate, and T-cell immune responses to adeno-associated virus vectors. Hum. Gene Ther. Methods *29*, 86–95.
26. Van Rooijen, N., and Sanders, A. (1994). Liposome mediated depletion of macrophages: mechanism of action, preparation of liposomes and applications. J. Immunol. Methods *174*, 83–93.
27. Lee, W.-Y., Moriarty, T.J., Wong, C.H.Y., Zhou, H., Strieter, R.M., van Rooijen, N., Chaconas, G., and Kubes, P. (2010). An intravascular immune response to *Borrelia burgdorferi* involves Kupffer cells and iNKT cells. Nat. Immunol. *11*, 295–302.
28. Karimipoor, M. (2019). Study break: recent advances in hemophilia gene therapy. Iran. Biomed. J. *23*, 7–8.
29. Mahlangu, J.N. (2018). Updates in clinical trial data of extended half-life recombinant factor IX products for the treatment of haemophilia B. Ther. Adv. Hematol. *9*, 335–346.
30. Borsotti, C., and Follenzi, A. (2018). New technologies in gene therapy for inducing immune tolerance in hemophilia A. Expert Rev. Clin. Immunol. *14*, 1013–1019.
31. Kubes, P., and Jenne, C. (2018). Immune responses in the liver. Annu. Rev. Immunol. *36*, 247–277.
32. Blaese, R.M., Culver, K.W., Miller, A.D., Carter, C.S., Fleisher, T., Clerici, M., Shearer, G., Chang, L., Chiang, Y., Tolstoshev, P., et al. (1995). T lymphocyte-directed gene therapy for ADA^- SCID: initial trial results after 4 years. Science *270*, 475–480.
33. Lukashev, A.N., and Zamyatnin, A.A., Jr. (2016). Viral vectors for gene therapy: current state and clinical perspectives. Biochemistry (Mosc.) *81*, 700–708.
34. Tsai, S.Y., Schillinger, K., and Ye, X. (2000). Adenovirus-mediated transfer of regulable gene expression. Curr. Opin. Mol. Ther. *2*, 515–523.
35. Hacein-Bey-Abina, S., Garrigue, A., Wang, G.P., Soulier, J., Lim, A., Morillon, E., Clappier, E., Caccavelli, L., Delabesse, E., Beldjord, K., et al. (2008). Insertional oncogenesis in 4 patients after retrovirus-mediated gene therapy of SCID-X1. J. Clin. Invest. *118*, 3132–3142.
36. Naldini, L., Blömer, U., Gallay, P., Ory, D., Mulligan, R., Gage, F.H., Verma, I.M., and Trono, D. (1996). In vivo gene delivery and stable transduction of nondividing cells by a lentiviral vector. Science *272*, 263–267.
37. Montini, E., Cesana, D., Schmidt, M., Sanvito, F., Ponzoni, M., Bartholomae, C., Sergi Sergi, L., Benedicenti, F., Ambrosi, A., Di Serio, C., et al. (2006). Hematopoietic stem cell gene transfer in a tumor-prone mouse model uncovers low genotoxicity of lentiviral vector integration. Nat. Biotechnol. *24*, 687–696.
38. Knolle, P.A., and Thimme, R. (2014). Hepatic immune regulation and its involvement in viral hepatitis infection. Gastroenterology *146*, 1193–1207.
39. Grieger, J.C., Soltys, S.M., and Samulski, R.J. (2016). Production of recombinant adeno-associated virus vectors using suspension HEK293 cells and continuous harvest of vector from the culture media for GMP FIX and FLT1 clinical vector. Mol. Ther. *24*, 287–297.
40. Jenne, C.N., Wong, C.H.Y., Petri, B., and Kubes, P. (2011). The use of spinning-disk confocal microscopy for the intravital analysis of platelet dynamics in response to systemic and local inflammation. PLoS One *6*, e25109.

OMTM, Volume *20*

Supplemental Information

Modulation of the liver immune microenvironment by the adeno-associated virus serotype 8 gene therapy vector

Agostina Carestia, Seok-Joo Kim, Franziska Horling, Hanspeter Rottensteiner, Christian Lubich, Birgit M. Reipert, Brian A. Crowe, and Craig N. Jenne

Supplemental Material

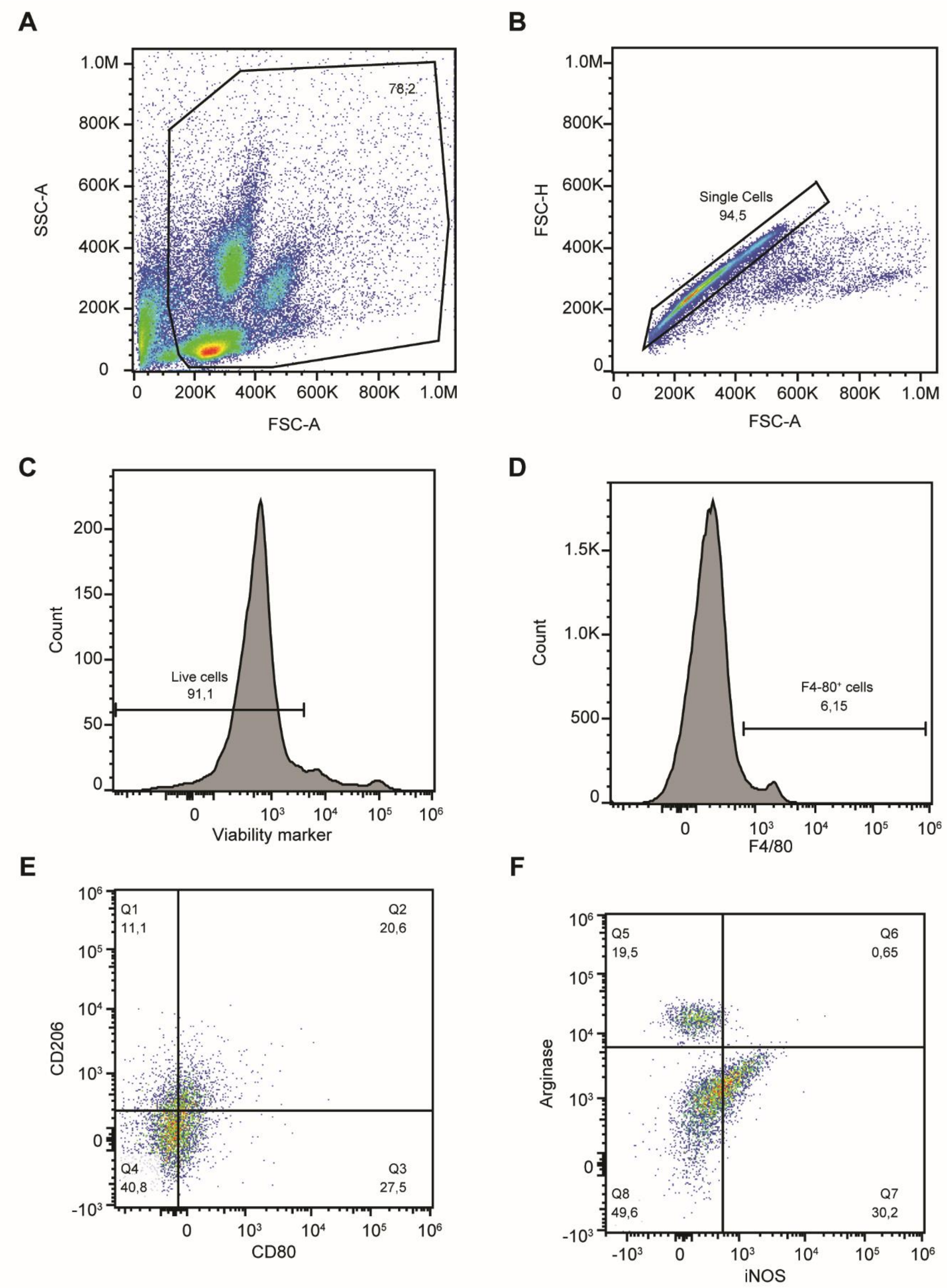

Supplemental Figure 1. Flow cytometry gating strategy used to determine macrophage polarization. After exclusion of debris **(A)** and doublets **(B)**, a live/dead staining was used to exclude dead cells **(C)**. Macrophages were identified on the expression of F4/80 **(D)**. For extracellular staining, a dot plot comparing CD80 and CD206 markers was used **(E)**. For intracellular staining, a dot plot comparing iNOS and Arginase was used

Supplemental Video 1. Viral capture by Kupffer cells and sinusoidal endothelium immediately following i.v. injection of AAV8.

Supplemental Video 2. Accumulation of virus on the surface of Kupffer cells and hepatocytes 30 min post i.v. injection of AAV8.

Supplemental Video 3. *In vivo* propidium iodide labelling of dead/damaged cells in the liver of control mice and AAV8 treated mice 1 day post-infection.

Supplemental Video 4. Imaging of platelet dynamics in the liver AAV8 treated mice 1 day and 3 days post-infection.

Supplemental Video 5. Tracking CD8+ cell interactions with liver Kupffer cells in control mice and at 28 days post AAV8 infection.
